# Deep Learning-Based Crowd Scene Analysis Survey

**DOI:** 10.3390/jimaging6090095

**Published:** 2020-09-11

**Authors:** Sherif Elbishlawi, Mohamed H. Abdelpakey, Agwad Eltantawy, Mohamed S. Shehata, Mostafa M. Mohamed

**Affiliations:** 1 The University of British Columbia, 3333 University Way, Kelowna, BC V1V 1V7, Canada; bishlawi@mail.ubc.ca (S.E.); mohamed.sami.shehata@ubc.ca (M.S.S.); 2 Memorial University of Newfoundland, St. John’s, NL A1C 5S7, Canada; mha241@mun.ca; 3 Electrical and Computer Engineering Department, University of Calgary, AB T2N 1N4, Canada; mostafa.mohamed@ucalgary.ca

**Keywords:** crowd scene, crowd counting, crowd action recognition, deep learning

## Abstract

Recently, our world witnessed major events that attracted a lot of attention towards the importance of automatic crowd scene analysis. For example, the COVID-19 breakout and public events require an automatic system to manage, count, secure, and track a crowd that shares the same area. However, analyzing crowd scenes is very challenging due to heavy occlusion, complex behaviors, and posture changes. This paper surveys deep learning-based methods for analyzing crowded scenes. The reviewed methods are categorized as (1) crowd counting and (2) crowd actions recognition. Moreover, crowd scene datasets are surveyed. In additional to the above surveys, this paper proposes an evaluation metric for crowd scene analysis methods. This metric estimates the difference between calculated crowed count and actual count in crowd scene videos.

## 1. Introduction

Automatic crowd scene analysis refers to investigating the behavior of a large group of people sharing the same physical area [1]. Typically, it counts the number of individuals per region, tracks the common individuals’ trajectories, and recognizes individuals’ behaviors. Therefore, automatic crowd scene analysis has many essential applications. It controls the spread of the COVID-19 virus [2] via ensuring physical distance between individuals in stores, parks, etc. Securing public events, such as sport championships [3], carnivals [4], new year celebrations [5], and Muslim pilgrimage [6], is another application of automatic crowd scene analysis. Crowd scene analysis supplies surveillance camera systems with the abiltiy to extract anomalous behaviors from a huge group of people [7,8,9]. Furthermore, analysis of crowd scenes of public places such as train stations, super stores, and shopping malls can show the effect of crowd path or the shortcomings of the design. Consequently, these studies can better safety considerations [10,11].

Due to the importance of analyzing crowd scenes, as illustrated above, different survey papers have been proposed. However, the existing survey papers either force traditional computer vision methods for crowd scenes analysis or review only one aspect of crowd analysis, such as crowd counting [12]. Therefore, this survey paper targets the provision of a comprehensive review of the evolution of crowd scene analysis methods up to the most recent deep learning [13] methods. This survey reviews the main two aspects of crowd analysis: (1) crowd counting and (2) crowd action recognition.

Additionally, this paper proposes an evaluation matrix, motivated by information theory, called crowd divergence (CD) for crowd scene analysis methods. In comparison with well-known evaluation matrices, e.g., mean squared error (MSE [14] and mean absolute error (MAE) [15], CD accurately measures how close the distribution of estimated crowd counts are to the actual distribution. In particular, the proposed metric calculates the amount of divergence between the actual and estimated counts.

The contribution of this paper is three-folds:

- surveying deep learning-based methods for crowd scenes analysis,
- reviewing available crowd scene datasets, and
- proposing crowd divergence (CD) for an accurate evaluation of crowd scenes analysis methods

The rest of this survey is organized as follows. Section 2 reviews the crowd counting method. In Section 3, crowd action recognition methods are surveyed. Section 4 reviews available crowd scene datasets. The novel crowd scene method evaluation matrix is proposed in Section 5. Section 6 provides a discussion of the paper. Section 7 concludes our survey paper and provides future directions.

## 2. Crowd Counting

Crowd counting refers to estimating the number of individuals who share a certain region. The following subsections review different methods that calculate how many individuals are in a physical region. For completeness, we start by reviewing traditional computer vision methods and then review deep learning-based methods.

### 2.1. Traditional Computer Vision Methods

#### 2.1.1. Detection-Based Approaches

Early approaches used detectors to detect peoples’ heads or shoulders in the crowd scene to count them, such as in [16,17]. Counting by detection is usually performed either in monolithic detection or parts-based detection. In monolithic detection, the detection is usually preformed based on pedestrian detection methods such as optical flow [18], histogram of oriented gradient (HOG) [19], Haar wavelets [20], edgelet [21], Particle flow [22], and shapelets [23]. Subsequently, the extracted features from the former detectors are fed into nonlinear classifiers such as Support Vector Machine (SVM) [24]; however, the speed is slow. A linear classifier such as linear SVM, hough forests [25], or boosting [26] usually provides a trade-off between speed and accuracy. Then, the classifier is slid over the whole image to detect candidates and to discard the less confident candidates. The results of sliding give the number of people in the scene.

The former methods cannot deal with the partial occlusion problem [27] when it is raised; therefore, part-based detection is adopted. Part-based detection focuses on body parts rather than the whole body such as the head and shoulders as in [17]. Part-based detection is more robust than monolithic, as reported in [17]. Based on 3D shapes [28], humans were modeled with ellipsoids, which was employed as a stochastic process [29] to calculate the number and shape configuration that best explains a segmented foreground object. Later on, Ge et. al [30] extended the same idea with Bayesian marked point process (MPP) [31] with a Bernoulli shape prototype [32]. Zhao et al. [33] used Markov chain Monte Carlo [34] to exploit temporal coherence for 3D human models across consecutive frames.

#### 2.1.2. Regression-Based Approaches

**Figure 1:**
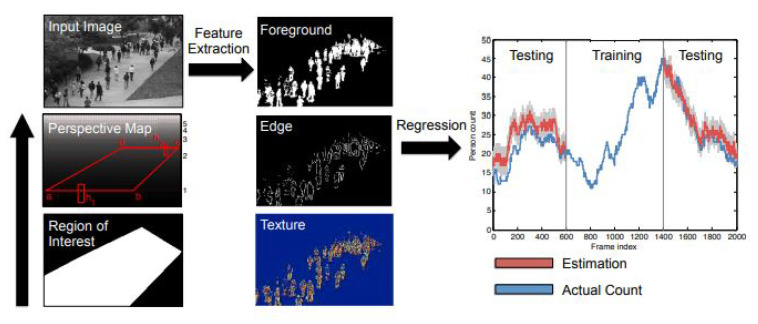
Crowd counting pipeline using regression model. Image from [47].

Although counting by detection or part-based approaches achieves reasonable results, it fails in very crowded scenes and under heavy occlusion. Counting by regression tries to mitigate the former problems. Typically, this method consists of two main components. The first component is extracting low-level features, such as Foreground features [35], texture [36], edge features [37], and gradient features [38]. The second component is mapping in a regression function, e.g., linear regression [39], piecewise linear regression [40], ridge regression [41], or Gaussian process regression, to map the extracted features into counts, as in [39]. The complete pipeline of this method is shown in Figure 1.

York et al. in [42] proposed a multi-feature method for accurate crowd counting. They aggregated different features, i.e., irregular and nonhomogeneous texture, Fourier interest points, head locations [43], and SIFT interest point [44], into one global feature descriptor. Then, this global descriptor was used in a multi-scale Markov Random Field (MRF) [45] to estimate counts. Moreover, the authors provided a new dataset (UCF-CC-50). Generally, regression-based approaches achieve good results, but they are based on a global count which results in a lack of spatial information.

#### 2.1.3. Density Estimation-Based Approaches

**Figure 2:**
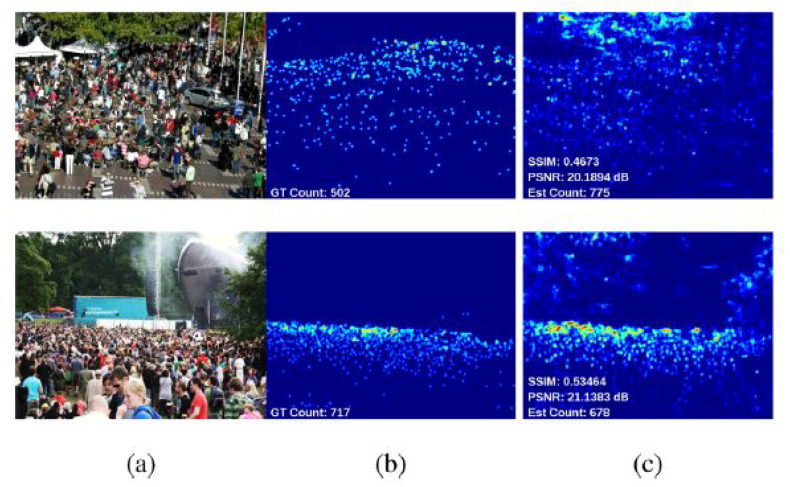
(**a**) Input image, (**b**) Ground truth, and (**c**) Estimated density maps. Image from [47].

These approaches build a density map to represent the number of individuals per region in an input image, as shown in Figure 2. In [46], the author built density maps via linearly mapping local patch features to its corresponding object. Formulating the problem in this way reduces the complexity of separating each object to count it and reduces the potential of counting errors in case of highly crowded scenes. Estimating the number of objects in this method equates to integration over local batches in the entire image.

In [48], the density map was built based on a loss function that minimizes the regularized risk quadratic cost function [48]. The solution was done by using cutting-plane optimization [49]. Pham et al. in [50] enhanced the work in [48] by learning nonlinear mapping. They used random forest regression [51] to vote for densities of multiple target objects. Moreover, their method reached real-time performance, and the embedding of subspaces formed by image patches was computed instead of mapping dense features and a density map.

Sirmacek et al. [52] proposed a scale and resolution-invariant method for density estimation. This method deploys Gaussian symmetric kernel functions [53] to calculate probability density functions (pdfs) [54] of different spots in consecutive frames. Finally, the number of people per spot is estimated via the value of the calculated pdfs. Table 1 summarize the main three categories of traditional crowd counting method.

### 2.2. Deep Learning Approaches

Convolutional Neural Networks (CNNs) are similar to plain Neural Networks (NNs) from the the perspective that they consist of neurons/receptive fields that have learnable weights and biases. Each receptive field receives a batch input and performs a convolution operation, and then, the result is fed into a nonlinearity function [55] (e.g., ReLU or Sigmoid). The input image to CNN is assumed to be an RGB image; therefore, the hidden layers learn rich features that contribute to the performance of the whole network (hidden layers and classifier). This structure has benefits in terms of speed and accuracy since the crowd scene images have lots of objects that need computationally expensive operations to detect. End-to-end networks mean the network takes the input image and directly produces the desired output.

The pioneering work with deep networks was proposed in [56]. An end-to-end deep convolutional neural network (CNN) regression model for counting people of images in extremely dense crowds was proposed. A collected dataset from Google and Flickr was annotated using a dotting tool. The dataset consists of 51 images, each of which has 731 people on average. The least number of counts in this dataset is 95, and the highest count is 3714. The network was trained on positive and negative classes. The positive images were labelled with the number of the objects, while the negative images were labelled with zero.

**Figure 3:**
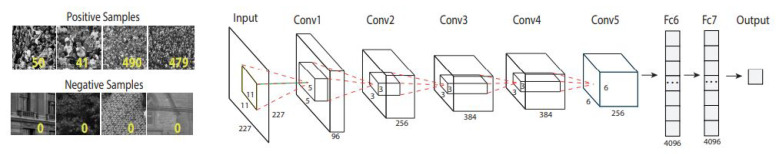
Convolutional Neural Network (CNN) architecture with positive and negative inputs. Image from [56].

Network architecture: This network consists of five convolutional layers and two fully connected layers. The network was trained on object classification with regression loss, as shown in Figure 3.

Another CNN-based approach following the former approach [57] proposed a real-time crowd density estimation method based on the multi-stage ConvNet [58]. The key idea in this method is based on assumption of some CNN connections being unnecessary; hence, similar feature maps from the second stage and their connections can be removed.

Network architecture: The network consists of two cascaded classifiers [59]; each classifier is multi-stage. The first stage consists of one convolutional layer in addition to a subsampling layer. The same architecture is used for the second stage. The last layer consists of a fully connected layer with five outputs to describe the crowd scene as either very low, low, medium, high, or very high. The feature maps from the first stage contribute only 1/7 of the total features; thus, the authors optimized this stage. The optimization was done based on measuring the similarity between maps. If the similarity is less than a predefined threshold, this map will be discarded to speed up the processing time.

**Figure 4:**
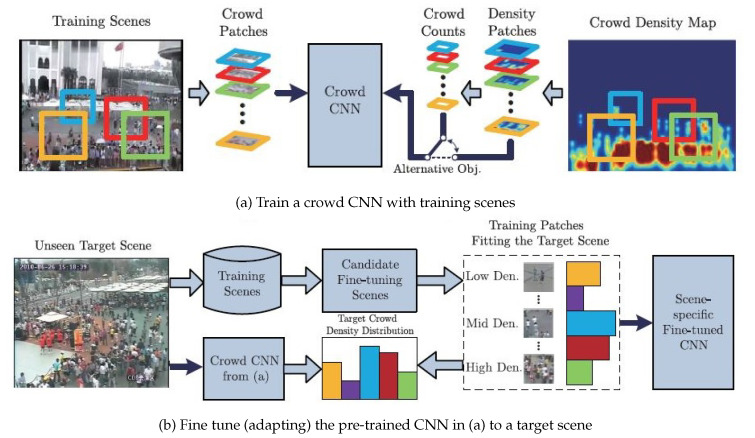
The internal structure of the cross-scene network with a fine-tuning scene module to generalize for unseen data. Image from [60].

**Figure 5:**
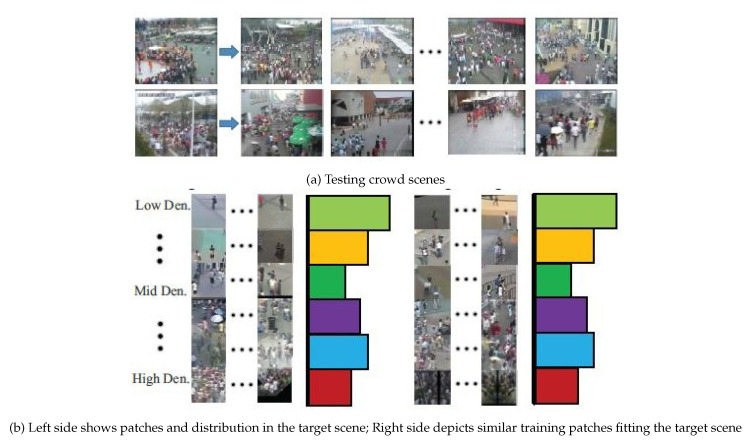
The nonparametric module. Image from [60].

In [60], the author observed that, when the trained network was applied on unseen data, the performance droped significantly. Consequently, a new CNN mechanism was trained on both crowd counts and density maps with switchable objectives, as shown in Figure 4. The nonparametric fine-tuning module is another contribution in this work. The main objective was to close the domain gap between the training data distribution and unseen data distribution. The nonparametric module consists of candidate scene retrieval, patch, and local patch retrieval. The main idea behind the candidate scene retrieval was retrieving training scenes that have similar perspective maps to the target scene from all training scenes. The local patch retrieval scene aims to select similar patches which have similar density distributions with those in the test scene, as shown in Figure 5.

Another framework to formulate the crowd scene uses generative adversarial network (GAN) [61]. In [62], the author provided two inputs to the network: the parent patch and the child patch. The parent patch is the whole image, while the child patch is 2 × 2 sub-patches. The idea behind this architecture is to minimize the cross scale consistency count between the parent and child patches.

Network architecture: The framework has two generators: parent $G_{large}$ and child $G_{small}$. The generator network G learns an end-to-end mapping from input crowd image patch to its corresponding density map with the same scale. Each generator consists of an encoder and a decoder [63], back to back, to handle scale variation.

In [64], the authors proposed two models for object and crowd counting. The first model is Counting CNN (CCNN), which learns how to map the image to its corresponding density map. The second model proposed is Hydra CNN, that can estimate object densities in very crowded scenes without knowing the geometric information of the scene.

**Figure 6:**
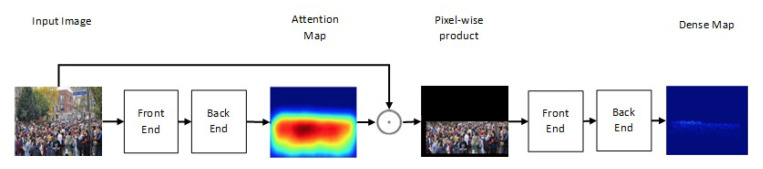
Structure of ADCrowdNet.

One of the newest state-of-the-art methods for accurate crowd counting came out in [65]. The authors proposed an attention-injective deformable convolutional network called ADCrowdNet that they claim can work accurately in congested noisy scenes. The network consists of two sections: Attention Map Generator (AMG) and Density Map Estimator (DME). AMG is a classification network that classifies the input image into crowd image or background image. The product of AMG is then used as input to DME to generate a density map of the crowd in the frame. This process is described in Figure 6. ADCrowdNet achieved the best accuracy for crowd counting on the ShanghaiTech dataset [66], UCF_CC_50 dataset [42], the WorldExpo’10 dataset [60], and the UCSD dataset [39]. In [67], Oh et al. proposed an uncertainty quantification method for estimating the count of the crowd. This method is based on a scalable neural network framework that uses a bootstrap ensemble. Method PDANet (Pyramid Density-Aware Attention-based network) [68] generates a density map representing the count of the crowd in each region of input images. This density map is generated by utilizing the attention paradigm, pyramid scale features, decoder modules for crowd counting, and a classifier to assess the density of the crowd in each input image. In DSSINet (Deep Structured Scale Integration Network) [69], structured feature representation learning and hierarchically structured loss function optimization are used to count the crowd. In [70], Reddy et al. tackled the problem of crowd counting by an adaptive few-shot learning. In [71], an end-to-end trainable deep architecture was proposed. This approach uses contextual information, generated by multiple receptive field sizes and learning the importance of each such feature at each image location, to estimate the crowd count in input images.

## 3. Crowd Action Recognition

In crowd analysis, recognizing different activities either for an individual or group of individuals is crucial for crowd safety. Therefore, this section focuses on reviewing crowd action recognition. Similar to the previous section, we start by reviewing traditional computer vision methods and then deep learning based methods for completeness to show how excellent deep learning methods are in this area.

### 3.1. Traditional Computer Vision Methods

One of the ways of examining crowd behavior was used in [72]. The authors proposed a way for detecting abnormal behavior from sensor data using a Hidden Markov Model [73], which is a statistical method based on a stochastic model used to model randomly changing systems.

In [74], the authors proposed a learning discriminative classifier from annotated 3D action cuboids to capture intra-class variation and sliding 3D search windows for detection. Then, a greedy k nearest neighbor algorithm [75] was used for automated annotation of positive training data.

In [76], the authors proposed a statistics-based approach for real-time detection of violent behaviors in a crowded scene. The method examines the change of the flow-vector magnitude over time and these changes are represented using a VIolent Flows (ViF) descriptor. The ViFs are then classified as violent or nonviolent behavior.

### 3.2. Deep Learning Approaches

**Figure 7:**
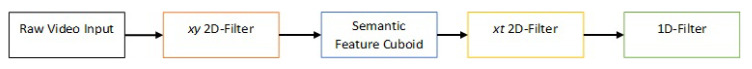
Single branch structure.

In [77], the authors provided the model in Figure 7 for capturing and learning dynamic representations of different objects in an image. The structure consists of four bunches of convolutional layers on xy-slices. Dimensions are then swapped using semantic feature cuboid so that xy becomes xt, followed by a bunch of xt convolutional layers. The last part of the network is a temporal layer to fuse cues learned from different xt-slices followed by fully connected layers.

Another big problem in crowd action recognition is recognizing semantic pedestrian attributes in surveillance images [78]. The authors proposed a Joint Recurrent Learning (JRL) model [78] for learning attribute context and correlation. The network utilizes Long short-term memory (LSTM) neural network for encoding and decoding. The intra-person attribute context of each person is modelled by the LSTM encoder. To make up for the poor image quality, the network uses auxiliary information from similar training images to provide inter-person similarity context. Lastly, LSTM decoder is constructed to model a sequential recurrent attribute correlation within the intra-person attribute context and the inter-person similarity context.

**Figure 8:**
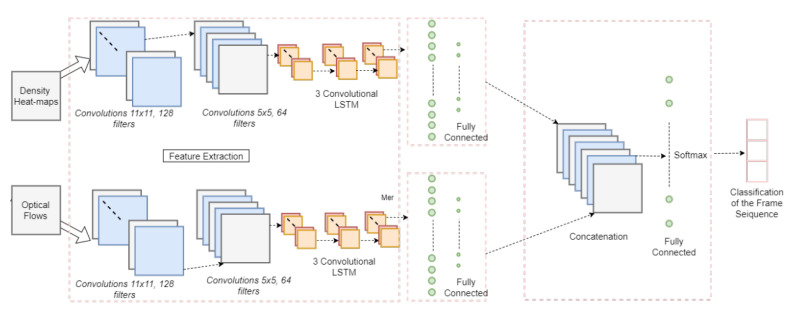
Abnormal event detection network from [79].

Detection of abnormal behavior in a crowded scene is a very promising research area that aims to prevent crimes before they happen. In [79], the authors proposed a model for abnormal event detection in a crowded scene. As Figure 8 shows, the model utilizes density heat maps and optical flow of the image frame. The network has two streams: one for density heat maps and one for optical flows of the frames. Both streams go through the same number of convolutional layers followed by fully connected layers, and then, the output of both streams are concatenated to output a classification of the frame sequence, thus detecting any abnormality.

**Figure 9:**
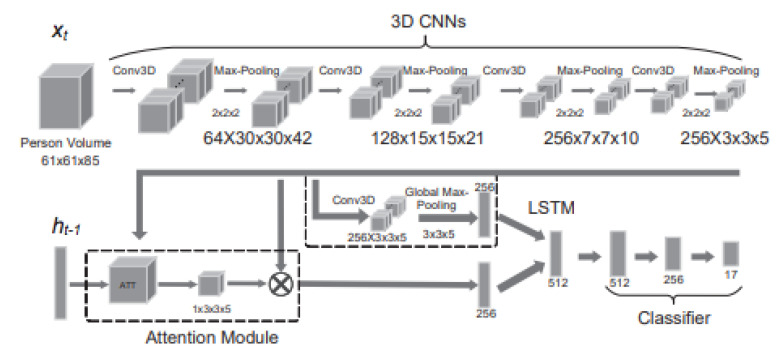
Action 4D attention neural network structure from [80].

One of the state-of-the-art methods for action recognition was proposed in [80]. The authors of the paper proposed a 4D model that recognizes actions using volumes of persons in the image. First, a people classification CNN was used to classify and detect every person in the image. Then, using the cropped image frame of each person, the volume of the person was used as input to the network Action4DNet shown in Figure 9. The input was convoluted multiple times in a 3D CNN; then, an attention model was used to learn the most relevant local sub-volume features, while max pooling was used to learn the global features. Both features were used as input to an LSTM network for action classification. Action 4D achieved very high accuracy compared to other evaluated models. However, in a scene with 10+ people, the accuracy went down because the network is dependent on having each person’s body clearly visible in the image. This shows that accurate action recognition in a crowd scene is still far from an achievable task in the current year. Table 2 compares crowd action recognition methods.

## 4. Crowd Scene Datasets

There are varieties of datasets, as shown Table 3, that can be used to train and/or evaluate crowd scene algorithms.

The most common one especially in deep learning algorithms is the ShanghaiTec dataset [66]. It has 1198 annotated images with internet images and street view images. WorldExpo’10 dataset [60] was created by 108 surveillance cameras that were monitoring Shanghai WorldExpo 2010. This dataset includes 1132 annotated video sequences.

The UCF dataset _CC_50 [42] has 50 annotated crowd frames. This dataset is considered one of the most challenging datasets due to the large variance in crowd counts and scenes. Typically, The crowd counts starts from 94 and can reach up to 4543.

UCSD dataset [39] consists of 2000 labelled images, each of size 158 × 238. The ground truth is labelled at the center of every object, and the maximum number of people is 46.

Mall [41] has various density levels. Moreover, it has various static and dynamic activity patterns.

There are datsets that are older but are still used in crowd scene counting such as Who do What at some Where (WWW) [85], UCLA [86], and Dyntex++ [87].

## 5. Crowd Divergence (CD)

Inspired by information theory [88] and the Kullback–Leibler equation [89], an evaluation matrix for crowd counting methods, i.e., Crowd Divergence (CD), was proposed. CD considers a crowd counting in consecutive frames as a density distribution. Hence, CD reveals how the predicted and the actual crowd counting distributions are close to each other over time.

Given a sequence of frames, CD calculates a divergence between the predicted and the actual crowd counting for each frame $x_{i}$. The divergence of frame $x_{i}$ is obtained via the following equation:

(1) $$S_{i}=t_{1}\left(x_{i}\right)\log\frac{t_{1}\left(x_{i}\right)}{t_{2}\left(x_{i}\right)},$$

where $t_{1}$ and $t_{2}$ are the actual and predicted crowd counts over time, respectively. To measure how the two distributions (i.e., predicted and actual crowd counts) are close to each other, CD sums up the scores $S_{i}$ over the sequence of frames, as follows:

(2) $$D_{KL(t_{1}\|t_{2})}=\sum_{i}S_{i}$$

It is worth mentioning that CD provides an evolution over time for crowd counting methods, whereas other evaluation metrics (e.g., Mean squared error, Mean absolute error, etc.) evaluate the predicted and the actual crowd counts of the last frame in a sequence.

## 6. Discussion

In this survey, we compared both traditional and deep learning methods for crowd counting and crowd action recognition. It turned out that deep learning-based approaches have high MAE and MSE compared to traditional-based approaches. One of the most important challenges is the lack of training dataset for different categories. One way to tackle this problem is (1) using data augmentation and applying scale changes augmentation and color changes and (2) using transfer learning to transfer the knowledge from a pretrained network to another (e.g., from the IMAGNET dataset [90] to the ShanghaiTec dataset [66]). A very important observation in crowd scene analysis is that CNN-based approach works very well; however, GAN networks such as in [62] have the highest performance in terms of MAE and MSE. Generative adversarial network (GAN) is a promising framework for crowd scene analysis, as shown in Table 4. Following GAN, the next context-aware method such as that in [91] achieves high performance.

## 7. Conclusions and Future Work

This paper surveys deep learning-based methods for crowd scene analysis. The surveyed methods are categorized into crowd counting and crowd action recognition. Crowd counting methods aim to estimate the number of individuals in a physical area. Crowd action recognition methods define the activity of a group of individual or a particular suspicious activity. For completeness, this survey reviews traditional computer vision methods for crowd scene analysis. It is evident that deep learning-based methods outperforms traditional computer vision methods in analyzing crowd scenes. Additionally, a novel performance metric, i.e., CD, is proposed to provide an accurate and robust evaluation of crowd scenes analysis method. This is achieved measuring the divergence between the actual trajectory/count and the predicted trajectory/count. Based on this survey, the GAN framework and context-aware are promising directions in crowd scene analysis.

## Figures and Tables

**Table 1:** Traditional Counting Approaches Comparison.

Traditional Counting Approaches	What They do	Pros and Cons
**Detection-based Approaches**	Use detectors to detect people’s heads and/or shoulders in the crowd scene	Reasonable results but fail in very crowded scenes and scenes with heavy occlusion
**Regression-based Approaches**	Low-level feature extraction and regression modeling	Good results but lack spatial information as they are based on global count
**Density Estimation-based Approaches**	Map input crowd image to its corresponding density map	Use spatial information to reduce counting errors

**Table 2:** Comparison of state-of-the-art crowd action recognation algorithms.

Method	Dataset	Underlying Technique
[72]	Street	Hidden Markov Model
[74]	CMU action detection dataset [81]	3D searching window with discriminative classifier
[76]	ASLAN [82]	statistics of how flow-vector magnitudes with SVM
[77]	WWW Crowd Dataset	CNN with xy-slices
[78]	PETA [83]	LTSM CNN
[79]	[84]	CNN with heat map and optical flow
[80]	4D action recognition dataset [80]	CNN for 4D model

**Table 3:** Datasets specifications.

Dataset	No. of Images	Resolution	Min	Ave	Max	Total Count
UCSD [39]	2000	158 × 238	11	25	46	49,885
Mall [41]	2000	320 × 240	13	-	53	62,325
UCF_CC_50 [42]	50	Varied	94	1279	4543	63,974
WorldExpo’10 [60]	3980	576 × 720	1	50	253	199,923
ShanghaiTech Part A [66]	482	Varied	33	501	3139	241,677
ShanghaiTech Part B [66]	716	768 × 1024	9	123	578	88,488

**Table 4:** Comparison of state-of-the-art crowd scene counting algorithms.

Ref.	Dataset											
	UCSD		Mall		UCF CC 50		WorldExpo ’10		Shanghai Tech-A		Shanghai Tech-B	
	MAE	MSE	MAE	MSE	MAE	MSE	MAE	MSE	MAE	MSE	MAE	MSE
[42]					468.0	590.3						
[92]	2.07	6.86	3.43	17.07								
[46]	1.7				493.4	487.1						
[50]	1.61	4.40	2.5	10.0								
[93]	1.98	1.82	2.74	2.10								
[94]	1.90	6.01	3.22	15.5								
[60]	1.60	3.31			467.0	498.5	12.9		181.8	277.7	32.0	49.8
[95]					452.5							
[66]	1.07	1.35			377.6	509.1	11.6		110.2	173.2	26.4	41.3
[96]	1.10		2.01		364.4							
[64]					333.7	425.2						
[91]					270.3		11.7					
[97]			2.75	13.4	361.7	493.3						
[98]					338.6	424.5			126.5	173.5	23.76	33.12
[99]	1.12	2.06			406.2	404.0	13.4					
[100]	2.86	13.0	2.41	9.12								
[12]					322.8	341.4			101.3	152.4	20.0	31.1
[101]	1.62	2.10			318.1	439.2	9.4		90.4	135.0	21.6	33.4
[62]	1.04	1.35			291.0	404.6	2.8		75.7	102.7	17.2	27.4
[65]	0.98	1.25			257.1	363.5	8.5		68.5	107.5	9.3	16.9

## References

[1] Musse S.R., Thalmann D. (1997). A model of human crowd behavior: Group inter-relationship and collision detection analysis. Computer Animation and Simulation’97.

[2] Watkins J. (2020). Preventing a Covid-19 Pandemic. https://www.bmj.com/content/368/bmj.m810.full.

[3] Jarvis N., Blank C. (2011). The importance of tourism motivations among sport event volunteers at the 2007 world artistic gymnastics championships, stuttgart, germany. J. Sport Tour..

[4] Da Matta R. (1991). Carnivals, Rogues, and Heroes: An Interpretation of the Brazilian Dilemma.

[5] Winter T. (2004). Landscape, memory and heritage: New year celebrations at angkor, cambodia. Curr. Issues Tour..

[6] Peters F.E. (1996). The Hajj: The Muslim Pilgrimage to Mecca and the Holy Places.

[7] Cui X., Liu Q., Gao M., Metaxas D.N. Abnormal detection using interaction energy potentials. Proceedings of the IEEE Conference on Computer Vision and Pattern Recognition (CVPR).

[8] Mehran R., Moore B.E., Shah M. (2010). A streakline representation of flow in crowded scenes. European Conference on Computer Vision.

[9] Benabbas Y., Ihaddadene N., Djeraba C. (2011). Motion pattern extraction and event detection for automatic visual surveillance. J. Image Video Process..

[10] Chow W.K., Ng C.M. (2008). Waiting time in emergency evacuation of crowded public transport terminals. Saf. Sci..

[11] Sime J.D. (1995). Crowd psychology and engineering. Saf. Sci..

[12] Sindagi V.A., Patel V.M. (2018). A survey of recent advances in cnn-based single image crowd counting and density estimation. Pattern Recognit. Lett..

[13] LeCun Y., Bengio Y., Hinton G. (2015). Deep learning. Nature.

[14] Wang Z., Bovik A.C. (2009). Mean squared error: Love it or leave it? a new look at signal fidelity measures. IEEE Signal Process. Mag..

[15] Willmott C.J., Matsuura K. (2005). Advantages of the mean absolute error (mae) over the root mean square error (rmse) in assessing average model performance. Clim. Res..

[16] Dollar P., Wojek C., Schiele B., Perona P. (2012). Pedestrian detection: An evaluation of the state of the art. IEEE Trans. Pattern Anal. Mach. Intell..

[17] Li M., Zhang Z., Huang K., Tan T. Estimating the number of people in crowded scenes by mid based foreground segmentation and head-shoulder detection. Proceedings of the 19th International Conference on Pattern Recognition (ICPR 2008).

[18] Brox T., Bruhn A., Papenberg N., Weickert J. (2004). High accuracy optical flow estimation based on a theory for warping. European Conference on Computer Vision.

[19] Dalal N., Triggs B. Histograms of oriented gradients for human detection. Proceedings of the Computer Vision and Pattern Recognition, CVPR 2005.

[20] Viola P., Jones M.J. (2004). Robust real-time face detection. Int. J. Comput. Vis..

[21] Wu B., Nevatia R. Detection of multiple, partially occluded humans in a single image by bayesian combination of edgelet part detectors. Proceedings of the 10th IEEE International Conference on Computer Vision (ICCV’05).

[22] Ali S., Shah M. A lagrangian particle dynamics approach for crowd flow segmentation and stability analysis. Proceedings of the 2007 IEEE Conference on Computer Vision and Pattern Recognition.

[23] Sabzmeydani P., Mori G. Detecting pedestrians by learning shapelet features. Proceedings of the Computer Vision and Pattern Recognition (CVPR’07).

[24] Chang C.C., Lin C.J. (2011). LIBSVM: A library for support vector machines. ACM Trans. Intell. Syst. Technol. (TIST).

[25] Gall J., Yao A., Razavi N., Van Gool L., Lempitsky V. (2011). Hough forests for object detection, tracking, and action recognition. IEEE Trans. Pattern Anal. Mach. Intell..

[26] Viola P., Jones M.J., Snow D. (2005). Detecting pedestrians using patterns of motion and appearance. Int. J. Comput. Vis..

[27] Zhang T., Jia K., Xu C., Ma Y., Ahuja N. Partial occlusion handling for visual tracking via robust part matching. Proceedings of the IEEE Conference on Computer Vision and Pattern Recognition.

[28] Kilambi P., Ribnick E., Joshi A.J., Masoud O., Papanikolopoulos N. (2008). Estimating pedestrian counts in groups. Comput. Vis. Image Underst..

[29] Whitt W. (2002). Stochastic-Process Limits: An Introduction to Stochastic-Process Limits and Their Application to Queues.

[30] Ge W., Collins R.T. Marked point processes for crowd counting. Proceedings of the IEEE Conference on Computer Vision and Pattern Recognition (CVPR 2009).

[31] Chatelain F., Costard A., Michel O.J. A bayesian marked point process for object detection: Application to muse hyperspectral data. Proceedings of the 2011 IEEE International Conference on Acoustics, Speech and Signal Processing (ICASSP).

[32] Juan A., Vidal E. Bernoulli mixture models for binary images. Proceedings of the 17th International Conference on Pattern Recognition (ICPR 2004).

[33] Zhao T., Nevatia R., Wu B. (2008). Segmentation and tracking of multiple humans in crowded environments. IEEE Trans. Pattern Anal. Mach. Intell..

[34] Geyer C.J. (1991). Markov Chain Monte Carlo Maximum Likelihood.

[35] Bouwmans T., Silva C., Marghes C., Zitouni M.S., Bhaskar H., Frelicot C. (2018). On the role and the importance of features for background modeling and foreground detection. Comput. Sci. Rev..

[36] Tuceryan M., Jain A.K. (1993). Texture analysis. Handbook of Pattern Recognition and Computer Vision.

[37] Mikolajczyk K., Zisserman A., Schmid C. (2003). Shape rEcognition With Edge-Based Features. https://hal.inria.fr/inria-00548226/.

[38] Hwang J.W., Lee H.S. (2004). Adaptive image interpolation based on local gradient features. IEEE Signal Process. Lett..

[39] Chan A.B., Liang Z.S.J., Vasconcelos N. Privacy preserving crowd monitoring: Counting people without people models or tracking. Proceedings of the IEEE Conference on Computer Vision and Pattern Recognition (CVPR 2008).

[40] Paragios N., Ramesh V. A mrf-based approach for real-time subway monitoring. Proceedings of the 2001 IEEE Computer Society Conference on Computer Vision and Pattern Recognition (CVPR 2001).

[41] Chen K., Loy C.C., Gong S., Xiang T. (2012). Feature mining for localised crowd counting. Proceedings of the British Machine Vision Conference.

[42] Idrees H., Saleemi I., Seibert C., Shah M. Multi-source multi-scale counting in extremely dense crowd images. Proceedings of the IEEE Conference on Computer Vision and Pattern Recognition.

[43] Vu T.H., Osokin A., Laptev I. Context-aware cnns for person head detection. Proceedings of the IEEE International Conference on Computer Vision.

[44] Lindeberg T. (2012). Scale Invariant Feature Transform. https://www.diva-portal.org/smash/get/diva2:480321/FULLTEXT02.

[45] Li S.Z. (2012). Markov Random Field Modeling in Computer Vision.

[46] Lempitsky V., Zisserman A. (2010). Learning to count objects in images. Advances in Neural Information Processing Systems, Proceedings of the Neural Information Processing Systems 2010, Vancouver, BC, Canada, 6 December 2010.

[47] Loy C.C., Chen K., Gong S., Xiang T. (2013). Crowd counting and profiling: Methodology and evaluation. Modeling, Simulation and Visual Analysis of Crowds.

[48] Teo C.H., Vishwanthan S., Smola A.J., Le Q.V. (2010). Bundle methods for regularized risk minimization. J. Mach. Learn. Res..

[49] Goffin J.L., Vial J.P. (2002). Convex nondifferentiable optimization: A survey focused on the analytic center cutting plane method. Optim. Methods Softw..

[50] Pham V.Q., Kozakaya T., Yamaguchi O., Okada R. Count forest: Co-voting uncertain number of targets using random forest for crowd density estimation. Proceedings of the IEEE International Conference on Computer Vision.

[51] Liaw A., Wiener M. (2002). Classification and regression by randomforest. News.

[52] Sirmacek B., Reinartz P. Automatic crowd density and motion analysis in airborne image sequences based on a probabilistic framework. Proceedings of the 2011 IEEE International Conference on Computer Vision Workshops (ICCV Workshops).

[53] Scaillet O. (2004). Density estimation using inverse and reciprocal inverse gaussian kernels. Nonparametric Stat..

[54] Cha S.H. (2007). Comprehensive survey on distance/similarity measures between probability density functions. City.

[55] Karlik B., Olgac A.V. (2011). Performance analysis of various activation functions in generalized mlp architectures of neural networks. Int. J. Artif. Intell. Expert Syst..

[56] Wang C., Zhang H., Yang L., Liu S., Cao X. (2015). Deep people counting in extremely dense crowds. Proceedings of the 23rd ACM International Conference on Multimedia.

[57] Fu M., Xu P., Li X., Liu Q., Ye M., Zhu C. (2015). Fast crowd density estimation with convolutional neural networks. Eng. Appl. Artif. Intell..

[58] Sermanet P., Kavukcuoglu K., Chintala S., LeCun Y. Pedestrian detection with unsupervised multi-stage feature learning. Proceedings of the IEEE conference on computer vision and pattern recognition.

[59] Sun Z., Wang Y., Tan T., Cui J. (2005). Improving iris recognition accuracy via cascaded classifiers. IEEE Trans. Syst. Man Cybern. Part Appl. Rev..

[60] Zhang C., Li H., Wang X., Yang X. Cross-scene crowd counting via deep convolutional neural networks. Proceedings of the IEEE Conference on Computer Vision and Pattern Recognition.

[61] Ledig C., Theis L., Huszár F., Caballero J., Cunningham A., Acosta A., Aitken A., Tejani A., Totz J., Wang Z. Photo-realistic single image super-resolution using a generative adversarial network. Proceedings of the IEEE Conference on Computer Vision and Pattern Recognition.

[62] Shen Z., Xu Y., Ni B., Wang M., Hu J., Yang X. Crowd counting via adversarial cross-scale consistency pursuit. Proceedings of the IEEE Conference on Computer Vision and Pattern Recognition.

[63] Badrinarayanan V., Kendall A., Cipolla R. (2017). Segnet: A deep convolutional encoder-decoder architecture for image segmentation. IEEE Trans. Pattern Anal. Mach. Intell..

[64] Onoro-Rubio D., López-Sastre R.J. (2016). Towards perspective-free object counting with deep learning. European Conference on Computer Vision.

[65] Liu N., Long Y., Zou C., Niu Q., Pan L., Wu H. Adcrowdnet: An attention-injective deformable convolutional network for crowd understanding. Proceedings of the IEEE Conference on Computer Vision and Pattern Recognition.

[66] Zhang Y., Zhou D., Chen S., Gao S., Ma Y. Single-image crowd counting via multi-column convolutional neural network. Proceedings of the IEEE Conference on Computer Vision and Pattern Recognition, Caesars Palace.

[67] Oh M.H., Olsen P.A., Ramamurthy K.N. Crowd counting with decomposed uncertainty. Proceedings of the Association for the Advancement of Artificial Intelligence (AAAI).

[68] Amirgholipour S., He X., Jia W., Wang D., Liu L. (2020). PDANet: Pyramid Density-aware Attention Net for Accurate Crowd Counting. arXiv Preprint.

[69] Liu L., Qiu Z., Li G., Liu S., Ouyang W., Lin L. Crowd counting with deep structured scale integration network. Proceedings of the IEEE International Conference on Computer Vision.

[70] Reddy M.K.K., Hossain M., Rochan M., Wang Y. Few-shot scene adaptive crowd counting using meta-learning. Proceedings of the IEEE Winter Conference on Applications of Computer Vision.

[71] Liu W., Salzmann M., Fua P. Context-aware crowd counting. Proceedings of the IEEE Conference on Computer Vision and Pattern Recognition.

[72] Andersson M., Rydell J., Ahlberg J. Estimation of crowd behavior using sensor networks and sensor fusion. Proceedings of the 12th International Conference on Information Fusion.

[73] Beal M.J., Ghahramani Z., Rasmussen C.E. The infinite hidden markov model. Proceedings of the Advances in Neural Information Processing Systems.

[74] Siva P., Xiang T. Action detection in crowd. Proceedings of the British Machine Vision Conference (BMVC).

[75] Li B., Yu S., Lu Q. (2003). An improved k-nearest neighbor algorithm for text categorization. arXiv.

[76] Hassner T., Itcher Y., Kliper-Gross O. Violent flows: Real-time detection of violent crowd behavior. Proceedings of the 2012 IEEE Computer Society Conference on Computer Vision and Pattern Recognition Workshops.

[77] Shao J., Loy C.C., Kang K., Wang X. Slicing convolutional neural network for crowd video understanding. Proceedings of the IEEE Conference on Computer Vision and Pattern Recognition.

[78] Wang J., Zhu X., Gong S., Li W. Attribute recognition by joint recurrent learning of context and correlation. Proceedings of the IEEE International Conference on Computer Vision.

[79] Lazaridis L., Dimou A., Daras P. Abnormal behavior detection in crowded scenes using density heatmaps and optical flow. Proceedings of the 2018 26th European Signal Processing Conference (EUSIPCO).

[80] You Q., Jiang H. Action4d: Online action recognition in the crowd and clutter. Proceedings of the IEEE Conference on Computer Vision and Pattern Recognition.

[81] Ke Y., Sukthankar R., Hebert M. Event detection in crowded videos. Proceedings of the 2007 IEEE 11th International Conference on Computer Vision.

[82] Kliper-Gross O., Hassner T., Wolf L. (2011). The action similarity labeling challenge. IEEE Trans. Pattern Anal. Mach. Intell..

[83] Deng Y., Luo P., Loy C.C., Tang X. Pedestrian attribute recognition at far distance. Proceedings of the 22nd ACM International Conference on Multimedia.

[84] Rabiee H., Haddadnia J., Mousavi H., Kalantarzadeh M., Nabi M., Murino V. Novel dataset for fine-grained abnormal behavior understanding in crowd. Proceedings of the 13th IEEE International Conference on Advanced Video and Signal Based Surveillance (AVSS).

[85] Péteri R., Fazekas S., Huiskes M.J. (2010). Dyntex: A comprehensive database of dynamic textures. Pattern Recognit. Lett..

[86] Fazekas S., Amiaz T., Chetverikov D., Kiryati N. (2009). Dynamic texture detection based on motion analysis. Int. J. Comput. Vis..

[87] Ghanem B., Ahuja N. (2010). Maximum margin distance learning for dynamic texture recognition. European Conference on Computer Vision.

[88] El Gamal A., Kim Y.H. (2011). Network Information Theory.

[89] Georgiou T.T., Lindquist A. (2003). Kullback-leibler approximation of spectral density functions. IEEE Trans. Inf. Theory.

[90] Russakovsky O., Deng J., Su H., Krause J., Satheesh S., Ma S., Huang Z., Karpathy A., Khosla A., Bernstein M. (2015). Imagenet large scale visual recognition challenge. Int. J. Comput. Vis..

[91] Shang C., Ai H., Bai B. End-to-end crowd counting via joint learning local and global count. Proceedings of the IEEE International Conference on Image Processing (ICIP).

[92] Chen K., Gong S., Xiang T., Change Loy C. Cumulative attribute space for age and crowd density estimation. Proceedings of the IEEE Conference on Computer Vision and Pattern Recognition.

[93] Wang Y., Zou Y. Fast visual object counting via example-based density estimation. Proceedings of the IEEE International Conference on Image Processing (ICIP).

[94] Xu B., Qiu G. Crowd density estimation based on rich features and random projection forest. Proceedings of the IEEE Winter Conference on Applications of Computer Vision (WACV).

[95] Boominathan L., Kruthiventi S.S., Babu R.V. (2016). Crowdnet: A deep convolutional network for dense crowd counting. Proceedings of the 24th ACM International Conference on Multimedia.

[96] Walach E., Wolf L. (2016). Learning to count with cnn boosting. European Conference on Computer Vision.

[97] Kumagai S., Hotta K., Kurita T. (2017). Mixture of counting cnns: Adaptive integration of cnns specialized to specific appearance for crowd counting. arXiv.

[98] Marsden M., McGuinness K., Little S., O’Connor N.E. (2016). Fully convolutional crowd counting on highly congested scenes. arXiv.

[99] Kang D., Ma Z., Chan A.B. (2018). Beyond counting: Comparisons of density maps for crowd analysis tasks—Counting, detection, and tracking. IEEE Trans. Circuits Syst. Video Technol..

[100] Sheng B., Shen C., Lin G., Li J., Yang W., Sun C. (2018). Crowd counting via weighted vlad on a dense attribute feature map. IEEE Trans. Circuits Syst. Video Technol..

[101] Sam D.B., Surya S., Babu R.V. Switching convolutional neural network for crowd counting. Proceedings of the IEEE Conference on Computer Vision and Pattern Recognition (CVPR).

